# Should scientists be allowed to bring distant human ancestors back to life?

**DOI:** 10.1371/journal.pbio.3003384

**Published:** 2025-09-04

**Authors:** Arthur L. Caplan

**Affiliations:** Division of Medical Ethics, NYU Grossman School of Medicine, New York, New York, United States of America

## Abstract

Recently, several attempts have been made to modify modern species with ancient DNA in the name of ‘de-extinction’. This Perspective argues that independent ethical oversight needs to be put in place before such efforts are extended to distant human ancestors and relatives.

## Teaser

Recent efforts by a private company to modify modern species with ancient DNA in the name of ‘de-extinction’ are both scientifically and morally suspect. A bright line requiring more than press releases as well as independent ethical oversight must be drawn before they are extended to distant human and hominid ancestors.

The recent trend for resurrecting extinct animals has made headlines globally and sparked controversy over the validity of the claims being made and the approach taken to reporting them. But, the bigger question should be, is this work ethical? And what if similar work was to be used to replicate features of historically distant human and hominid ancestors?

Much publicity accompanied the announcement by the Dallas-based biotechnology company Colossal Biosciences, that it had successfully ‘de-extincted’ dire wolves and has plans to do so for other species. However, once the actual genetic techniques involved are examined (Box 1), the claims of species de-extinction are far from credible. What has been done and is being planned is actually the creation of hybrids somewhat akin to but far from identical to extinct animal species. Still, despite the inability to actually de-extinct any species, private efforts to modify ancient DNA in the name of de-extinction are likely to continue.

**Box 1. The technology of de-extinction**

De-extinction describes the process by which species that have entirely disappeared can be recreated. Cloning and, more recently, genetic engineering through CRISPR, are the two major techniques for attempting species restoration.

Using cloning alone for de-extinction is at best a means of trying to preserve nearly extinct animals and organisms. Cloning can and has been used to preserve animals nearing extinction. But, since cloning requires living sources from adult cells and their eggs that are not available for animals that have gone totally extinct, its utility in the service of de-extinction is severely limited. At best, it offers a last-ditch tool to prevent imminent extinction. Also, many cloned animals exhibit health issues and suffer premature deaths, thus limiting the role cloning can play in species preservation [1].

Gene editing via CRISPR also has limitations [2,3]. The first step in using CRISPR for de-extinction is sequencing the extinct animal’s genome. Scientists also need to procure the genome of a very close living relative, if one exists. If the sequence of the genome of the extinct animal is known, and if the genome of a very close living relative is available, the differences can be mapped. Once the traits deemed characteristic of the extinct animal are identified, CRISPR is used to modify the living relative’s genome [2,3]. This creates a new genome for a hybrid animal that is far from a replica of the extinct animal. Cloning follows with implantation into a surrogate.

Using CRISPR in this way has huge drawbacks. Ancient DNA is very fragmented, having been broken down over time by bacteria, exposure to UV light and other agents, making locating all ancient genes difficult. Introducing many genetic changes into a background genome creates unknowable and possibly dangerous interactions between preexisting and newly introduced genes and the proteins and traits they create.

Furthermore, existing surrogate mothers may not create a close replica of the environment the extinct animal’s mother’s womb and diet would have created. And duplicating any resulting animal’s diet and social upbringing is next to impossible since these are unknown, making de-extinction implausible.

Once the limits of CRISPR recreation are understood, proponents of de-extinction tend, perhaps not surprisingly, to point toward environmental benefits, not actual species recreation, as their rationale. Ecosystems that depended on keystone species have lost the diversity they once supported. As environmental change occurs, Colossal Biosciences and other supporters of creating hybrid creatures say they may be useful in restoring ecological balance [4].

A prime example is the wooly mammoth. Four thousand years ago, the tundras of Russia and Canada consisted of a rich grass and ice-based ecosystem. Today they are melting. A few dozen changes to the genome of modern elephants—to give them subcutaneous fat, woolly hair, and sebaceous glands—might suffice to create a variation that is functionally similar to the mammoth. Returning this keystone-like species to the tundras could stave off some effects of warming, as ‘de-extincted mammoths’ could keep the region colder by eating dead grass, thus enabling the sun to reach spring grass, whose deep roots prevent erosion. They could also increase reflected light by felling trees, which absorb sunlight, and punch through insulating snow so that freezing air penetrates the soil [4]. However, this rationale is not convincing, leaving the ethics of the effort in doubt [5].

In a world that is rapidly overheating, the idea that modified elephants could rebalance any ecosystem is unpersuasive on its face. The number of healthy animals needed, the time to create them, and the time for herds in novel climates to have an impact are enormous roadblocks to undertaking the effort. And the unknown impact of CRISPR engineering on the well-being of hybrid, mammoth-like elephants in terms of their diet, risk of infectious diseases, social needs, climate change, and overall health from CRISPR, surrogacy and cloning (Box1) make moral defenses of announcements misdescribed as de-extinction exceedingly ethically dubious.

Following their recent announcement, Colossal Biosciences said it was “proud to return the dire wolf to its rightful place in the ecosystem,” suggesting the same ecological rationale offered for recreating mammoths. But this argument makes no sense. Dire wolves have been extinct for thousands of years, and the ecology that supported them and their role or benefit for future ecological stability are not known. Yet despite the justification of ecological restoration for modifying wolves to resemble dire wolves being weak, Colossal Bioscience has repeated it in pointing toward a planned effort to restore the 600-year extinct flightless bird, the moa.

Others have noted that animal de-extinction efforts made them morally uneasy. The resurrection might not be good for the animals. Where would they live, give the loss of their former habitat, and possible harm from predators or poachers? Would they be lonely? What would they be fed [5]? The animal welfare concerns about these creatures are legitimate and ought to be fully addressed in any publications and media announcements [1].

The effort and the drive for publicity for the inaccurately described ‘de-extinction’ work and the constant targeting of new species raise another huge issue that has not drawn attention—what if a privately held company were to use CRISPR-based techniques aimed at ‘restoring’ extinct human ancestors?

Three hundred thousand years ago, at least nine species of hominids were alive [6]. Today, only *Homo sapiens* remains. Theories abound around the disappearance of these other species, from *H. sapiens* having better infant survival rates to *H. sapiens* hunting other species or interbreeding with them and simply assimilating their genetics. Modifying a contemporary human genome through tracking ancestral hominid DNA might provide some answers. To do so would require locating DNA from fossils, which would be possible for Neanderthals among other ancestors [6]. Proponents might insist that using genes from *Homo neanderthalensis* or other extinct ancestors’, such as Denisovans, using the CRISPR-driven techniques Colossol Biosciences used to create so-called dire wolves could answer further questions.

Although such efforts are currently only theoretical, the decision about de-extincting or, more accurately, partially reconstructing, human ancestors needs to be addressed now before any effort is made. Ancient hominid de-extinction should not be left in the hands of private, closely held, for-profit companies. Whether an effort at ancient hominid partial recreation is justified should be debated and regulated by an international body with the power to hold public debates, offer independent risk assessments, and insist on standards for undertaking such experiments and protection for any potential surrogates involved. Expertise needs to be sought from appropriate independent scientific experts concerning the welfare of beings created in this manner and the potential dangers to human surrogates. Penalties for any unsanctioned attempts must be put in place. Investors in such efforts must be held strictly liable for any harm caused by such an undertaking. The media must demand not just a press release but documentation of appropriate independent ethical approval and oversight.

The de-extinction of human predecessors could be undertaken at any time by private entitities. Given how recent efforts have been overly hyped, lack a persuasive rationale, have had little independent peer review, and have taken place with unethical indifference to animal welfare, this seems highly undesirable. The mere possibility merits much more than responding after the fact with ethical questions. A strong case exists for proactive ethical debate and transparent regulatory oversight of experiments with all extinct species.
